# The serum tenascin C level is a marker of metabolic disorder-related inflammation affecting pancreatic cancer prognosis

**DOI:** 10.1038/s41598-024-62498-x

**Published:** 2024-05-26

**Authors:** Katsuhiko Sato, Hayato Hikita, Minoru Shigekawa, Kazumasa Soma, Ryohei Yamauchi, Jihyun Sung, Seiya Kato, Yoichi Sasaki, Shinnosuke Kudo, Kenji Fukumoto, Kumiko Shirai, Kazuhiro Murai, Yuki Tahata, Teppei Yoshioka, Akira Nishio, Yoshinobu Saito, Takahiro Kodama, Yutaka Sasaki, Tomohide Tatsumi, Tetsuo Takehara

**Affiliations:** 1 Department of Gastroenterology and Hepatology, Osaka University Graduate School of Medicine, 2-2, Yamadaoka, Suita City, Osaka Japan; 2 Osaka Central Hospital, 3-3-30, Umeda, Kitaku, Osaka City, Osaka Japan

**Keywords:** Pancreatic cancer, Cancer prevention

## Abstract

Obesity is a risk factor for pancreatic cancer development, partly due to the tissue environment of metabolic disorder-related inflammation. We aimed to detect a tissue environment marker triggered by obesity-related metabolic disorders related to pancreatic cancer progression. In murine experiments, Bl6/j mice fed a normal diet (ND) or a high-fat diet (HFD) were orthotopically injected with mPKC1, a murine-derived pancreatic cancer cell line. We used stocked sera from 140 pancreatic cancer patients for analysis and 14 colon polyp patients as a disease control. Compared with ND-fed mice, HFD-fed mice exhibited obesity, larger tumors, and worse prognoses. RNA sequencing of tumors identified tenascin C (TNC) as a candidate obesity-related serum tissue environment marker with elevated expression in tumors of HFD-fed mice. Serum TNC levels were greater in HFD-fed mice than in ND-fed mice. In pancreatic cancer patients, serum TNC levels were greater than those in controls. The TNC-high group had more metabolic disorders and greater CA19-9 levels than did the TNC-low group. There was no relationship between serum TNC levels and disease stage. Among 77 metastatic patients treated with chemotherapy, a high serum TNC concentration was an independent poor prognostic factor. Pancreatic cancer patients with high serum TNC levels experienced progression more rapidly.

## Introduction

Obesity and a high-fat diet (HFD) are risk factors for pancreatic cancer development in murine models, and the tissue environment of obesity and HFD-induced inflammation may be key factors. Genetically engineered Kras mutant mice fed an HFD exhibit accelerated pancreatic cancer progression from pancreatic intraepithelial neoplasia to invasive cancer via cyclooxygenase 2-related inflammation and alterations in metabolic pathways, including beta-oxidation^1,2^. Obesity itself enhances inflammation and fibrosis in the pancreas via enhanced cholecystokinin expression in islet cells and promotes pancreatic cancer progression, which is impeded by body weight loss^3^. Obese patients with a body mass index (BMI) greater than 35 kg/m^2^ have worse survival than those with a BMI less than 25 kg/m^2^, showing that obesity may accelerate pancreatic cancer development and lead to a poor prognosis^4^. These reports suggest that the inflammatory environment triggered by obesity and HFD feeding may promote pancreatic cancer development.

Obesity-related metabolic disorders and concomitant inflammation may be key factors promoting pancreatic cancer. Although obesity and HFD feeding-induced inflammation have been shown to promote pancreatic cancer progression in murine models^1,3^ and human data show that obesity is a risk factor for human pancreatic cancer development^1,3,5,6^, few studies have focused on the inflammatory progression of pancreatic cancers triggered by obesity and related metabolic disorders, namely, dyslipidemia, hyperuricemia, hypertension, and diabetes mellitus. Obesity-related metabolic disorders are often described as metabolic syndrome^7^. Inflammation triggered by metabolic syndrome worsens multiple diseases in different parts of the body, including cardiovascular disease and metabolic dysfunction-associated steatohepatitis (MASH)^7^, and pancreatic cancer development may also be promoted by these diseases. In clinical practice, the cachexic state triggered by pancreatic cancer induces body weight loss^8^. As a result, body weight and blood chemistry data at diagnosis, the only time point when accurate data can be obtained, may underestimate obesity and underlying metabolic disorders. Reactions to metabolic dysfunction, including obesity, differ strongly among individuals. For example, 85% of obese patients in the United States do not have diabetes^9^, and not all obese patients have fatty liver^10^. A BMI over 30 kg/m^2^, a described risk factor for pancreatic cancer^5^, is quite rare in the Japanese population. However, the number of pancreatic cancer patients is increasing, and many have obesity-related metabolic disorders, such as hypertension, dyslipidemia, and diabetes mellitus. Thus, a serum marker for the inflammatory tissue environment triggered by obesity-related metabolic disorders may allow for the stratification of patients at high risk for metabolic disorder-related pancreatic cancer progression, regardless of the presence of obesity.

In this study, we aimed to detect a serum tissue environment marker for obesity-induced inflammation in a murine pancreatic cancer model and to analyze its relationship with the inflammatory tissue environment triggered by obesity-related metabolic disorders in humans.

## Results

### HFD-fed mice had greater body weights, higher serum nonesterified fatty acid (NEFA) levels, and worse prognoses than did ND-fed mice in an immunocompetent orthotopic pancreatic cancer mouse model

**Figure 1:**
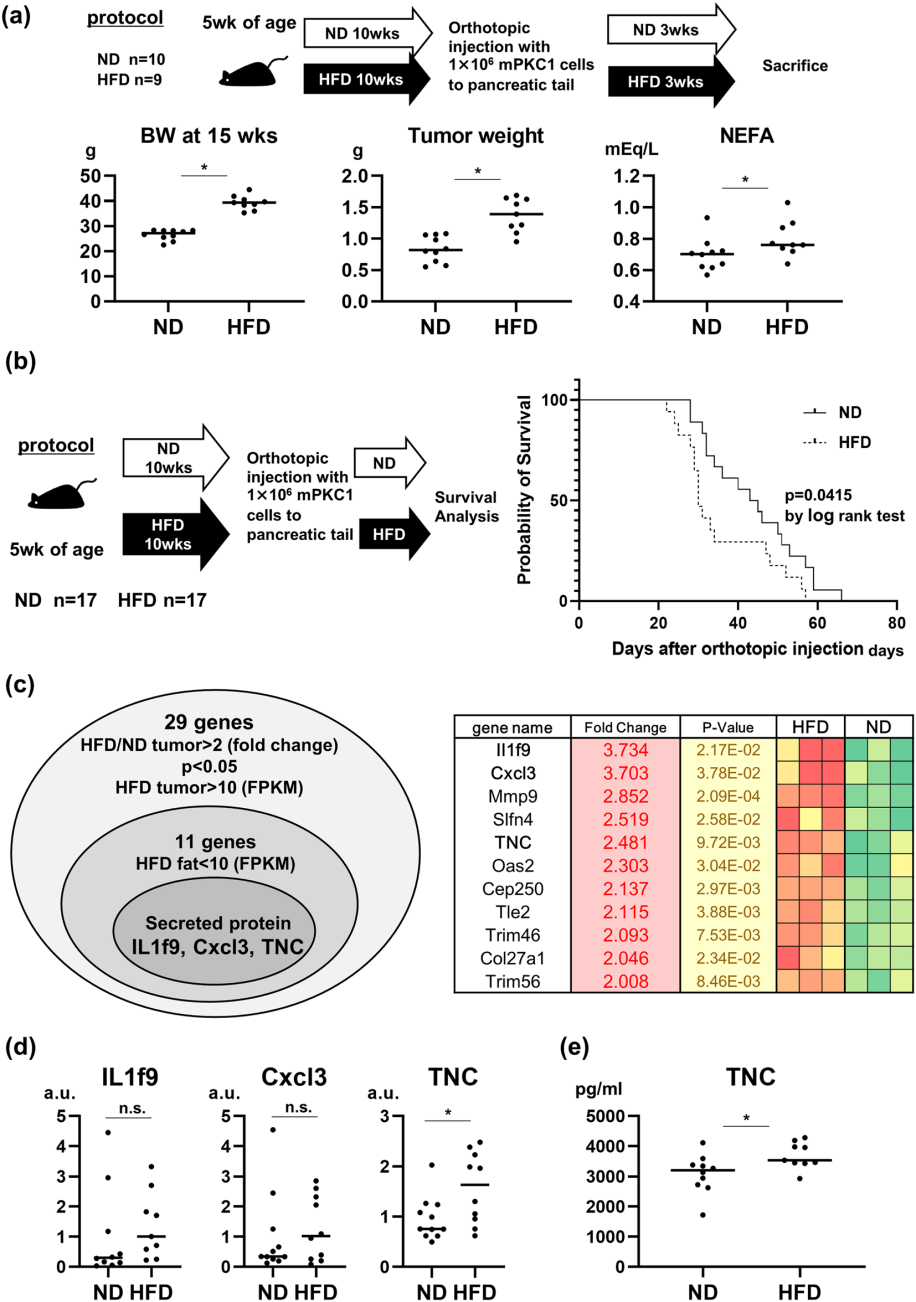
RNA sequencing of tumors from high-fat diet (HFD)-fed mice and normal diet (ND)-fed mice revealed serum tenascin C as a candidate serum marker for obesity-related development of murine orthotopic pancreatic tumors. Mice fed an ND or an HFD from 5 weeks of age to 15 weeks of age were orthotopically injected with murine-derived mPKC1 pancreatic cancer cells. (**a**) Scheme of the protocol for orthotopic injection of ND- and HFD-fed mice (ND: n = 10, HFD: n = 9). Body weight (BW) at 15 weeks of age, tumor weight 3 weeks after orthotopic injection, and serum nonesterified fatty acid (NEFA) levels 3 weeks after orthotopic injection of ND- and HFD-fed mice. (**b**) Survival curve of ND- and HFD-fed mice after orthotopic injection (log rank test). (**c**) RNA sequencing of tumors from ND- and HFD-fed mice was performed. Among twenty-nine genes with FPKM values that were 2 times greater in HFD-fed tumors than in ND-fed tumors and FPKM > 10 in HFD-fed tumors, 11 genes with FPKM values below 10 in adipose tissues are shown. Three genes (IL1f9, Cxcl3, TNC) encode secreted proteins according to the Human Protein Atlas (www.proteinatlas.org). (**d**) Real-time PCR of tumors from ND- and HFD-fed mice. (**e**) Serum TNC levels in ND- and HFD-fed mice. Mann‒Whitney U test; bar only, median; *, *p* < 0.05.

C57Bl6/J mice were raised on an ND or an HFD for 10 weeks beginning at 5 weeks of age. Orthotopic injection of mPKC1 cells into the pancreatic tail was performed at 15 weeks of age. These mice were continuously fed a previously fed diet, either an ND or an HFD, and analyzed 3 weeks after orthotopic injection (Fig. 1a). Compared with ND-fed mice, HFD-fed mice had greater body weights, greater tumor weights, and greater serum NEFA levels (Fig. 1a). According to the prognosis analysis, HFD-fed mice had a worse prognosis than did ND-fed mice (Fig. 1b).

### RNA sequencing of tumors from HFD-fed mice and ND-fed mice revealed serum tenascin C (TNC) as a candidate for HFD-related serum prognostic markers

To detect tumor-derived HFD feeding-related serum markers, we performed RNA sequencing of orthotopic tumors from ND- and HFD-fed mice. To minimize the effect of contamination of adipose tissue in the pancreatic tumors of HFD-fed mice, we simultaneously performed RNA sequencing of the perigonadal fat of HFD-fed mice and compared the results. Among the 396 genes whose fragments per kilobase of exon per million mapped reads (FPKM) in the tumors of HFD-fed mice were 2 times greater than those in the tumors of ND-fed mice, 29 genes had FPKM values greater than 10 in the tumors of HFD-fed mice (Fig. 1c). To reduce the effect of contamination of adipose tissues in RNA extraction due to the large amount of fat attached to pancreatic tumors in HFD-fed mice, we excluded genes with FPKM values greater than 10 in the adipose tissue of HFD-fed mice. Among the 11 genes, 3 candidate markers, namely, Il1f9, Cxcl3, and tenascin C (TNC), were secreted proteins according to the Human Protein Atlas (www.proteinatlas.org) (Fig. 1c). Increased mRNA expression of TNC in the tumors of HFD-fed mice compared with the tumors of ND-fed mice was observed (Fig. 1d). Serum TNC levels were greater in HFD-fed mice than in ND-fed mice (Fig. 1e). Serum TNC levels correlated with tumor weight but not with body weight at orthotopic injection or with serum NEFA levels 3 weeks after orthotopic injection (Supplementary Fig. 1).

### Serum TNC levels were greater in pancreatic cancer patients than in controls, and TNC levels correlated with the inflammatory markers neutrophil-to-lymphocyte ratio (NLR) and albumin and the tumor marker carbohydrate antigen 19–9 (CA19-9)

**Figure 2:**
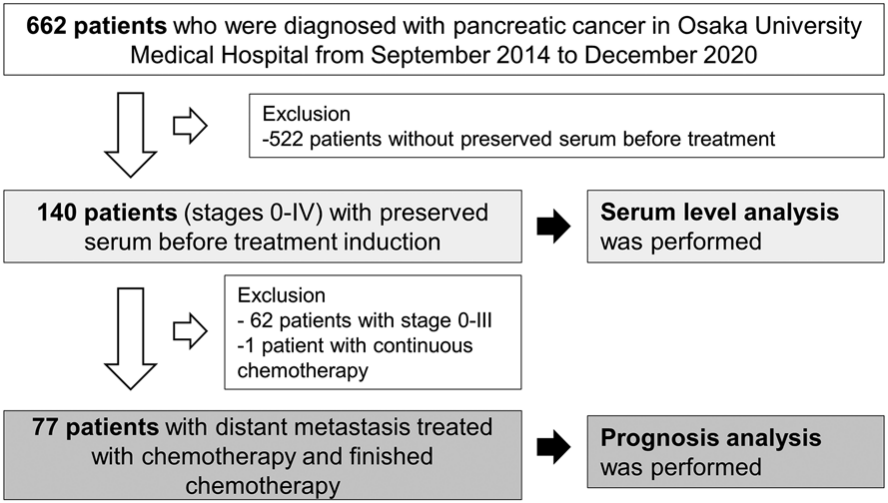
Pancreatic cancer patients enrolled in this study.

**Figure 3:**
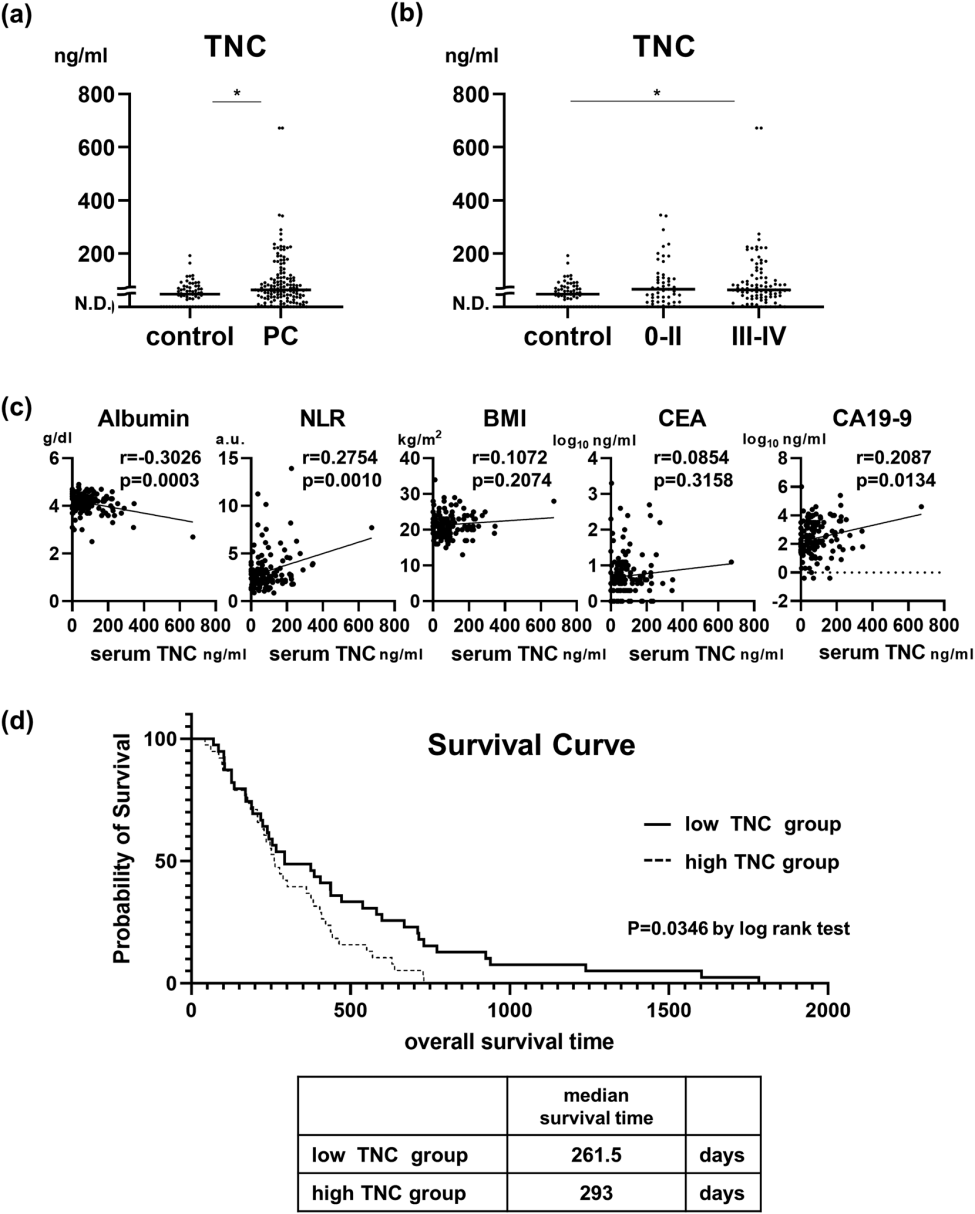
TNC is a prognostic factor that correlates with the albumin concentration, neutrophil-to-lymphocyte ratio and carbohydrate antigen 19–9 (CA19-9) concentration. (**a**) Serum TNC levels of pancreatic cancer patients and healthy controls (control, n = 64; stage 0-IV, n = 140; Mann‒Whitney U test). (**b**) Serum TNC levels and trend analysis of pancreatic cancer patients and healthy controls staged according to the UICC 8th edition. *, *p* < 0.05. (control, n = 64; stage 0-II, n = 50; stage III-IV, n = 90; *p* = 0.003, Jonckheere-Terpstra trend test). (**c**) Patients with stage 0-IV disease were analyzed (n = 140). Pearson’s correlation coefficients for TNC and albumin concentrations, the neutrophil-to-lymphocyte ratio, body mass index, CEA level, and CA19-9 level were calculated. (**d**) Survival curves of 77 patients with metastatic pancreatic cancer in the TNC-low and TNC-high groups according to the Kaplan‒Meier method (TNC-low group: n = 39; TNC-high group: n = 38; log rank test). *, *p* < 0.05.

In total, 662 consecutive patients were diagnosed with pancreatic cancer at Osaka University Medical Hospital between September 2014 and December 2020 (Fig. 2). Five hundred twenty-two patients without preserved serum were excluded, and 140 patients with preserved serum and informed consent were analyzed. In our cohort of 140 stage 0-IV pancreatic cancer patients, there were 78 males and 62 females. The median age was 71.2 years. There were 50 metabolic disorder-free patients with hypertension, hyperuricemia, dyslipidemia, and diabetes mellitus (Table 1). The median serum TNC concentration was 64.0 ng/mL for stage 0-IV patients, which was significantly greater than that for healthy controls (Fig. 3a). The healthy controls were younger than pancreatic cancer patients (supplementary Table 1). There was a positive trend according to stage progression in serum TNC levels in control, stage 0–II, and stage III–IV pancreatic cancer patients (Fig. 3b). Analysis of stage 0–IV patients revealed that serum TNC levels were weakly correlated with the inflammatory markers albumin and NLR and the tumor marker CA19-9 but not with the body composition marker BMI or the tumor marker carcinoembryonic antigen (CEA) (Fig. 3c). We then divided our cohort into two groups according to the median TNC level. No difference was observed in the frequency of individual metabolic disorders, including hypertension, dyslipidemia, hyperuricemia, and diabetes mellitus, between the TNC low and TNC-high groups. However, the percentages of metabolic disorder-free patients with hypertension, hyperuricemia, dyslipidemia, and diabetes were greater in the TNC-low group than in the TNC-high group (Table 1). The TNC-high group had higher serum CA19-9 levels than did the TNC-low group, but no differences in stage, albumin level, NLR, BMI or CEA level were observed (Table 1).

**Table 1:** Baseline characteristics of stage 0-IV pancreatic cancer patients and univariate analysis of the TNC low group and high group.

variables		All patients (n = 140)	TNC low group (n = 70)	TNC high group (n = 70)	*p* value
Sex	Male/female	78/62	40/30	38/32	0.734
Age, years	Median (range)	71.2 (41.2–87.4)	69.5 (41.2–84.2)	72 (43–87.3)	0.268
BMI, kg/m^2^	Yes/no	21.3 (13.3–33.9)	21.2 (15.7–33.9)	21.3 (13.3–29.4)	0.744
ASA-PS, class	Class 0-I/II-III	22/118	14/56	8/62	0.164
Smoking	Yes/no	57/83	28/42	29/41	0.863
Alcohol	Yes/no	72/68	34/36	38/32	0.499
none of below	Metabolic disorder/none	90/50	39/31	51/19	0.034
Hypertension	Yes/no	52/88	26/44	26/44	1.000
Dyslipidemia	Yes/no	36/104	16/54	20/50	0.439
Hyperuricemia	Yes/no	14/126	5/65	9/61	0.260
Diabetes mellitus	Yes/no	62/78	28/42	34/36	0.394
Tumor size, mm	Median (range)	27 (0–80)	28 (0–80)	25.5 (0–70)	0.307
Location of tumor	Head/body or tail	58/82	27/43	31/39	0.493
Obstructive jaundice	Yes/no	22/118	8/62	14/56	0.164
stage I/I/II/III/IV	0/I/II/III/IV	6/4/41/11/78	3/2//20/6/39	3/2/21/5/39	0.919
Hb, mg/dL	Median (range)	13 (8.1–16.59	13.0 (8.1–16.5)	13.1 (8.9–16)	0.614
Neutrophil-to-lymphocyte ratio	Median (range)	2.7 (0.9–13.9)	2.54 (0.90–11.27)	2.74 (0.87–13.94)	0.293
Platelet-to-lymphocyte ratio	Median (range)	138.1 (60.5–365.0)	144.7 (60.5–355.6)	130.2 (60.6–365.4)	0.990
PNI	Median (range)	49.7 (32.2–62.0)	50.4 (36.8–62.0)	49.2 (32.2–60.3)	0.264
Amylase, U/L	Median (range)	78.5 (24–1305)	79 (32–229)	76.5 (24–130.5)	0.993
Albumin, g/dL	Median (range)	4.2 (2.5–4.9)	4.2 (3–4.9)	4.2 (2.5–4.7)	0.251
HbA1C, percentage	Median (range)	6.2 (3.3–13)	6.15 (4.9–13)	6.4 (3.3–12.8)	0.437
CEA, ng/mL	Median (range)	4 (1–1850)	4 (1–1850)	4.5 (1–487)	0.090
CA19-9, U/mL	Median (range)	164.8 (0.4–94,000)	113 (0.4–940,000)	396 (0.4–247,600)	0.028

TNC, tenascin C, BMI, body mass index; ASA PS, American Society of Anesthesiologists physical status; Hb, hemoglobin; PNI, prognostic nutritional index; CEA, carcinoembryonic antigen; CA19-9, carbohydrate antigen.

### A high serum TNC concentration was an independent predictor of poor prognosis in patients with metastatic pancreatic cancer treated with chemotherapy

To assess the impact of serum TNC levels on prognosis, we analyzed 77 metastatic pancreatic cancer patients who began and finished chemotherapy among the 140 patients (Fig. 3). There were 41 males and 36 females, and the median age was 69 years. The median follow-up period was 277 days (Supplementary Table 2). When comparing the TNC-low group and TNC-high group among the 77 patients divided by the same cutoff value of 64.0 ng/mL, the CA19-9 level was greater in the TNC-high group than in the TNC-low group (Supplementary Table 2). According to the univariate Cox hazard model, the serum TNC concentration was a prognostic factor. Multivariate analysis of age, CEA, and CA19-9 revealed that the serum TNC concentration was an independent prognostic factor for metastatic pancreatic cancer patients (Table 2). The median survival time of patients in the TNC-low group was longer than that of patients in the TNC-high group (293 days vs. 261.5 days, *p* = 0.0346, by the log rank test) (Fig. 3d).

**Table 2:** Prognostic factors for metastatic pancreatic cancer patients.

Variable				Univariate analysis			Multivariate analysis	
		n	HR	95% C.I	*p* value	HR	95% C.I	*p* value
Sex	Male	36	1.14	0.723–1.789	0.578			
	Female	41	1					
Age	≥ 75 yrs	20	1.37	0.820–2.295	0.229	1.76	1.022–3.048	0.464
	< 75 yrs	57	1			1		
BMI	≥ 21.2	38	1.02	0.647–1.601	0.938			
	< 21.2	39						
ASA-PS	class 2–3	44	0.96	0.611–1.518	0.872			
	class 0–1	33	1					
Tumor size	≥ 30 mm	45	1.23	0.766–1.988	0.387			
	< 30 mm	32	1					
metabolic disorder	yes	32	0.87	0.551–1.384	0.564			
	none	45	1					
Neutrophil-to-lymphocyte ratio	≥ 2.84	38	1.53	0.965–2.395	0.071			
	< 2.84	39	1					
Platelet-to- lymphocyte ratio	≥ 144.6	38	1.11	0.706–1.745	0.650			
	< 144.6	39	1					
PNI	≥ 48.8		0.811	0.516–1.274	0.364			
	< 48.8		1					
Amylase	≥ 72 IU/L	38	0.78	0.489–1.257	0.312			
	< 72 IU/L	39	1					
Albumin	≥ 3.5 g/dL	38	0.72	0.459–1.134	0.157			
	< 3.5 g/dL	39	1					
HbA1C	≥ 6.5%	33	1.25	0.789–1.987	0.341			
	< 6.5%	42	1					
CEA	≥ 6 ng/mL	38	1.34	0.845–2.117	0.215	1.22	0.738–2.021	0.436
	< 6 ng/mL	39	1			1		
CA19-9	≥ 1202 U/mL	38	1.48	0.934–2.340	0.095	1.34	0.806–2.223	0.260
	< 1202 U/mL	39	1			1		
TNC	≥ 64.0	38	1.67	1.032–2.688	0.037	1.70	1.020–2.831	0.042
	< 64.0	39	1			1		

HR, hazard ratio; C.I., confidence interval; BMI, body mass index; ASA-PS, American Society of Anesthesiologists physical status; PNI, prognostic nutritional index; CEA, carcinombryonic antigen; CA19-9, carbohydrate antigen 19–9; TNC, tenascin C.

## Discussion

TNC may be a serum marker for metabolic disorder-related inflammation affecting pancreatic cancer development. Although TNC levels were not correlated with BMI according to our human data, we showed that compared with patients with low serum TNC levels, pancreatic cancer patients with high serum TNC levels were more likely to have at least one metabolic disorder, including hypertension, dyslipidemia, hyperuricemia, or diabetes mellitus. The proinflammatory state triggered by metabolic syndrome has been shown to increase the risk of cardiovascular disease, diabetes mellitus, and MASH syndrome^7^. In our human cohort of stage 0-IV pancreatic cancer patients, the serum TNC concentration was positively correlated with the NLR and negatively correlated with the ALB concentration, indicating that TNC levels are related to inflammation (Fig. 3c). Although pancreatic cancer itself triggers progressive inflammation via cachexia, which is affected by disease severity^11^, our data revealed no difference in either stage or tumor size between the TNC-high group and the TNC-low group (Table 1). These findings indicate that TNC may be a serum tissue environment marker for metabolic disorder-related inflammation and may promote pancreatic cancer development, which is least affected by tumor severity.

In our study, TNC was detected in the RNA sequences of tumors from HFD- and ND-fed mice in which tumor cells were the dominant population. HFD-fed mice with larger tumors had higher levels of serum TNC than did ND-fed mice (Fig. 1), and tumor size correlated with serum TNC levels (Supplementary Fig. 1). In in vitro experiments, tumor-derived TNC has been reported to enhance pancreatic cancer progression via resistance to apoptosis^12^ and to promote invasion^13^. The activation of MET in pancreatic tumor cells enhanced TNC expression, and the paracrine TNC-mediated crosstalk between cancer cells and stellate cells promoted a tumor-progressive microenvironment^14^. Breast cancer cell-derived TNC enhances the activity of the NOTCH and WNT pathways and the metastatic niche, promoting pulmonary metastasis^15^. Considering the poor prognosis of the TNC-high group compared with the TNC-low group in metastatic pancreatic cancers (Fig. 3c), tumor-derived TNC itself may play a role in the cancer progression niche of the primary tumor and metastatic sites, which may worsen the prognosis of metastatic pancreatic cancer patients.

It must be noted, however, that our study has limitations. First, our study was retrospective, and to clarify the relationships between metabolic syndrome, pancreatic cancer progression, and TNC counts, a prospective study with precancer body weight and precancer TNC counts is needed. Second, the reaction to metabolic load differs among individuals, making clarification of the role of TNC in metabolic disorder-induced pancreatic cancer progression difficult. Third, our cohort included only one patient with a BMI over 30 kg/m^2^, which is often described as a pancreatic cancer risk factor. Further studies are needed to clarify the role of obesity and related metabolic disorders in pancreatic cancer progression.

In conclusion, we showed that the serum TNC level, which may reflect the obesity-related serum tissue environment in HFD-fed mice, is a metabolic disorder-related inflammatory marker affecting the prognosis of pancreatic cancer patients. In clinical practice, high serum TNC levels in patients with metabolic disorders may promote pancreatic cancer progression, regardless of obesity status, and more strict surveillance may be needed to predict poor prognosis.

## Methods

### Animal experiments

Five-week-old male C57BL6/J mice were purchased from Charles River Laboratories Japan (Charles River Laboratories Japan, Yokohama, Japan). These mice were fed either an ND or an HFD (HFD 32, CLEA Japan, Inc., Tokyo, Japan) for 10 weeks. At 15 weeks of age, the mice were injected with 5*10^5^ mPKC1 cells in the pancreatic tail, which were eluted in 25 µl of Matrigel (356,230, Corning, Corning, NY). mPKC1 cells are a murine-derived pancreatic cancer cell line with a C57B6/J background that we previously cultured from the pancreatic tumors of *Kras*^*LSL G12D*^* ElaCre trp53*^*fl/fl*^* EYFP*^*Tg/Tg*^ mice^16^. All mice were continuously fed a previously fed diet, either an ND or an HFD, after orthotopic injection for further analyses. The survival, body weight, and serum NEFA data of HFD-fed mice orthotopically injected were compared with those of HFD-fed sham mice not orthotopically injected in our previous study, but no data from ND-fed mice were used in the previous study^17^. Furthermore, no analysis concerning the comparison of ND- and HFD-fed mice has been performed or reported^17^. The mice were euthanized by cervical dislocation. For invasive procedures including cervical dislocation, 4 mg/kg midazolam (at a concentration of 400 µg/ml), 5 mg/kg butorphanol (at a concentration of 500 µg/ml), and 0.75 mg/kg medetomidine (at a concentration of 75 µg/ml) were intraperitoneally injected at a volume of 10 µl per gram body weight. After orthotopic injection, 0.75 mg/kg medetomidine was introduced to reverse the anesthesia. Tissues were fixed with 4% paraformaldehyde. The animals were kept on a 12-h/12-h light/dark cycle and provided food and water ad libitum. All animal experiments were approved by the Committee of Experimental Animal Science of Osaka University and conducted within its regulations (approval number: 30–015). All animal experiments were carried out according to the Guidelines for Proper Conduct of Animal Experiments, and the methods used were reported in accordance with the ARRIVE guidelines.

### Real-time PCR

RNA was extracted from pancreatic tumors from orthotopic model mice with an RNeasy kit (#74106, Qiagen, Hilden, Germany). Reverse transcription was performed with PrimeScript reverse transcriptase (#2680, TaKaRa, Shiga, Japan). A gene expression assay by real-time PCR was performed with thunderbird probe qPCR mix (QPS-101, Toyobo, Osaka, Japan). A QuantiStudio 6 Flex real-time PCR system (Thermo Fischer Scientific) was used according to the manufacturers’ protocol. The primers used for the TaqMan assays for TNC (Mm00495662_m1), Cxcl3 (Mm-01701838_m1), and IL1f9 (Mm00463327_m1) were obtained from Thermo Fisher Scientific (Thermo Fisher Scientific, Waltham, MA, USA).

### Enzyme-linked immunosorbent assay (ELISA)

A Mouse TNC/Tenascin C ELISA Kit (LS-F74808-1, LifeSpan Biosciences, Seattle, WA) was used to measure the serum TNC concentration in the mice. A human Tenascin C ELISA kit (ab277081, Abcam, Cambridge, UK) was used to measure the TNC concentration in human serum. An NEFA C test kit (Wako, #279-75401; Fujifilm Wako Pure Chemical Corporation, Osaka, Japan) was used to measure serum NEFA concentrations in both mice and humans.

### RNA sequencing

RNA was extracted from orthotopic tumors of both ND- and HFD-fed mice and perigonadal fat of HFD-fed mice as described above. RNA sequencing was performed and analyzed using these samples as previously described^18^. The RNA sequencing data were deposited in the DNA Data Bank of Japan (Bioproject Accession number: PRJDB17381).

### Clinical data

Pancreatic cancer patients diagnosed at Osaka University Medical Hospital between September 2014 and December 2020 were identified from clinical conference records. All patients enrolled were pathologically diagnosed with pancreatic cancer and underwent multidisciplinary expert consultation. Patients who provided informed consent for serum preservation and whose serum was preserved prior to treatment induction, including chemotherapy and surgery, were enrolled. Tumors were staged according to the UICC 8th edition. We described the location of the pancreatic tumor within the pancreas as the head, body, or tail. For prognosis-related analyses, data from metastatic pancreatic cancer patients who had finished chemotherapy were obtained. All patients were followed until death or transfer to hospice. Patients were considered to have hypertension, dyslipidemia, or hyperuricemia when they had been prescribed medication at the time of diagnosis. Patients were considered to have diabetes mellitus if they had been prescribed medication at the time of diagnosis or had an HbA1c level greater than 6.5% at diagnosis. The levels of reported inflammatory markers, including the NLR^19–22^, platelet-to-lymphocyte ratio^23,24^, and prognostic nutritional index^25,26^, were calculated from laboratory data at diagnosis. The median was used as the cutoff value for laboratory data-related variables, including TNC counts. Within 343 individuals who had medical check-up at Osaka central hospital from April 2023 to May 2023 and agreed to preserve serum, 64 individuals without previous diagnosis of any disease and whose BMI was between 18.5 and 25 kg/m^2^ and considered normal according to Japan Society for the Study of Obesity were enrolled as healthy controls. All patients agreed to serum preservation after providing informed consent. This study followed the Declaration of Helsinki and was approved by the Institutional Review Board of Osaka University (Approval Number: 17160 and 17032).

### Statistical analysis

Continuous variables were analyzed with the Mann‒Whitney U test. Categorical variables were analyzed with the chi-square test. The Kaplan‒Meier method was used to estimate the overall survival of patients in the TNC-high group and the TNC-low group. The log-rank test was used to evaluate the survival curve. A Cox proportional hazard model was used for prognosis-related analyses. Correlations between TNC levels and related variables were analyzed by the Pearson correlation coefficient. The Jonckheere-Terpstra trend test was used for trend analysis to compare the stages of pancreatic cancer patients. JMP Pro 17 (SAS Institute, Inc., Tokyo, Japan) was used for all the statistical analyses. We considered a *p* value less than 0.05 to indicate statistical significance.

### Ethical approval

This study followed the Declaration of Helsinki and was approved by the Institutional Review Board of Osaka University (Approval Number: 17160 and 17032).

### Supplementary Information

Supplementary Table 1.

Supplementary Table 2.

Supplementary Figure 1.

## Data Availability

The datasets generated during and/or analyzed during the current study are not publicly available for privacy reasons but are available from the corresponding author upon reasonable request. RNA sequence data that support the findings of this study have been deposited in the DNA data bank of Japan with the primary accession code PRJDB17381.
