# Supplementary figures and images for: The serum tenascin C level is a marker of metabolic disorder-related inflammation affecting pancreatic cancer prognosis
Source: Sci Rep. 2024 May 26;14:12028. doi: 10.1038/s41598-024-62498-x (PMC11128447; doi:10.1038/s41598-024-62498-x)

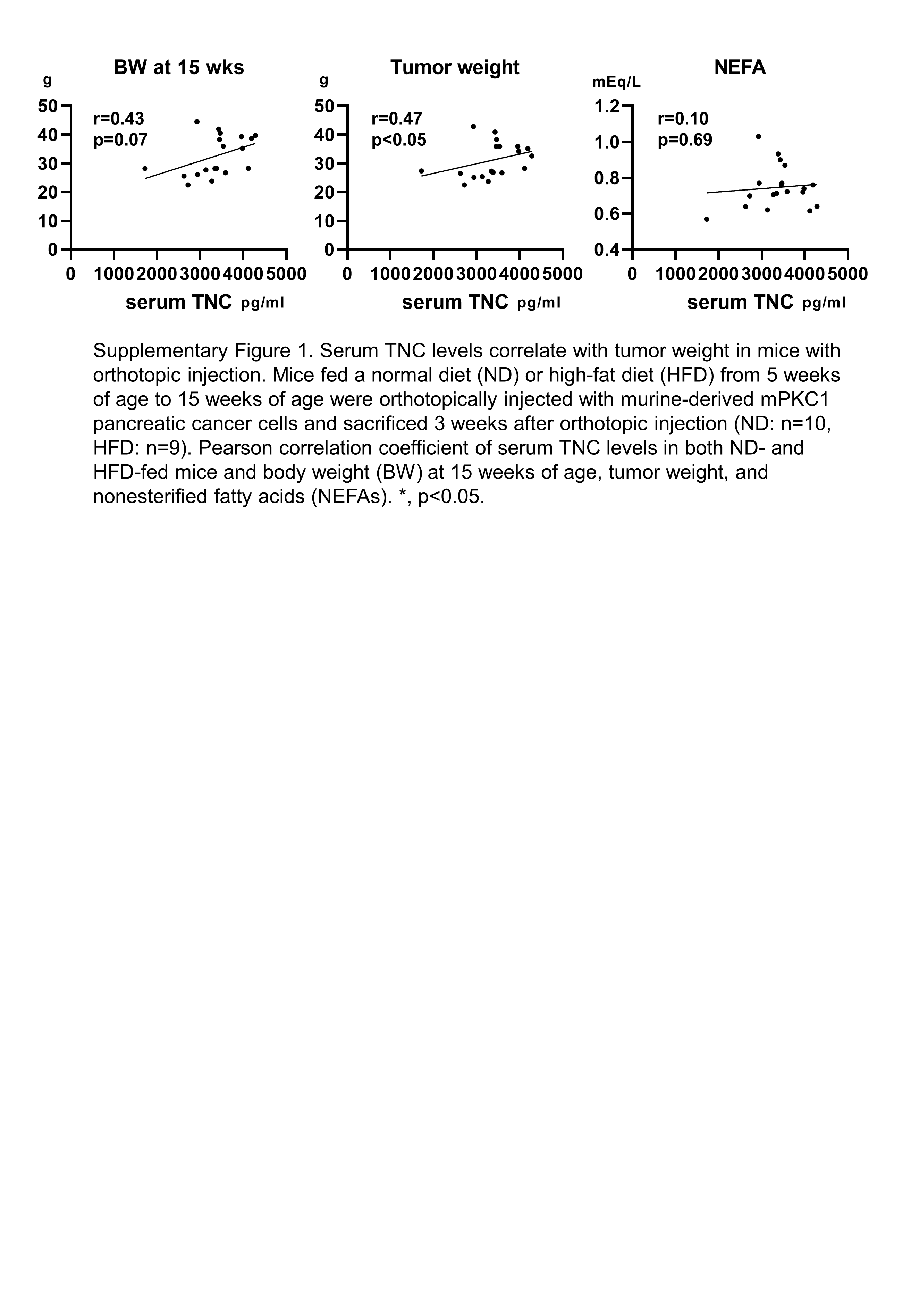

Supplement: Supplementary file 3 — Supplementary Figure 1. [file 41598_2024_62498_MOESM3_ESM.tif]
